# Market assessment to improve fibre recycling within the EU textile sector

**DOI:** 10.1177/0734242X231178222

**Published:** 2023-06-14

**Authors:** Emanuel Boschmeier, Wolfgang Ipsmiller, Andreas Bartl

**Affiliations:** Institute of Chemical, Environmental and Bioscience Engineering, TU Wien, Vienna, Austria

**Keywords:** Fibre recycling, challenges, recycled fibres, market situation, economic boundary conditions, legislative measures, policies

## Abstract

Clothing is one of the primary human needs, but today’s business models turned most apparel into a disposable product. As a matter of fact, the rising demand results in the production of Millions of tons of textile waste every year which is either landfilled, incinerated or exported, with only small amounts being recycled. One promising recycling attempt towards a circular economy in the apparel sector is fibre-to-fibre recycling, where end-of-use clothes serve as input material for the production of new fibres and, eventually, new apparel. In this work, together with fashion brands and a textile research organisation, a mapping of the market situation and the economic boundary conditions regarding textile fibre recycling are presented. Generally, fibre-to-fibre recycling technologies need more public attention and intensive research, and development is necessary as well as legislative instruments that encourage interest in textile recycling. The market situation for recycled fibres is promising and will tend to an increased demand in recycled fibres in the future. Mandatory certification ensures a sustainable product and fast fashion should be held back. Textile waste landfilling, export regulations as well as sustainable lifestyle education shall be considered by EU legislature to ensure that recycling materials are actually used and create a market pull for textile waste back into the industry.

## Introduction

### The economy

**Figure 1.**
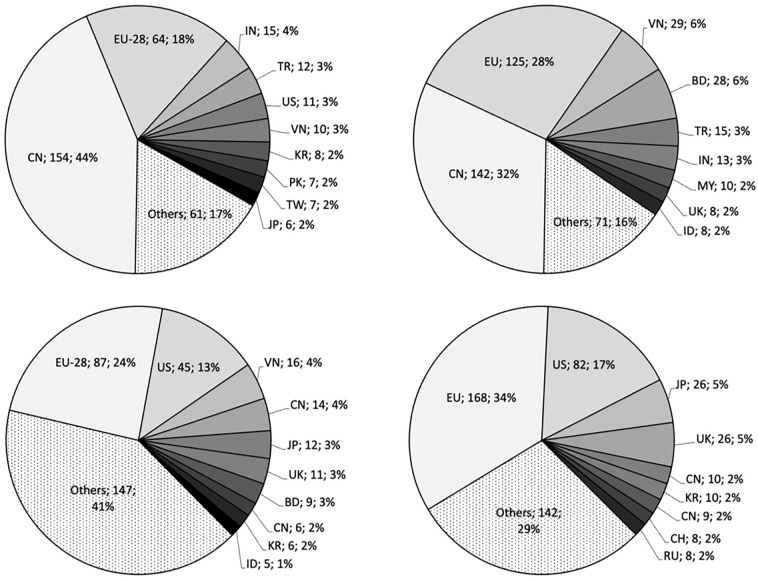
Top exporters (first line) and importers (second line) of textiles (left column) and apparel (right column) in 2020 in Billion US$ and respective shares; Country abbreviations according to ISO 3166-1 (WTO, 2021).

The textile and apparel market are of eminent importance, both globally and in Europe. Without a doubt, Asia, and above all China, is the most important player in the apparel and textile sector. As shown in Figure 1, China is accounting for 44% (i.e. 154 billion US$) of global textile and 32% (i.e. 142 billion US$) of global apparel exports. Amongst the top ten exporters of apparel and textiles, one can see eight Asian countries. However, the EU apparel and textile industry is of considerable size and ranks second after China in both textiles (87 billion US$, i.e. 24% share) and apparel (125 billion US$, i.e. 28% share).

**Figure 2.**
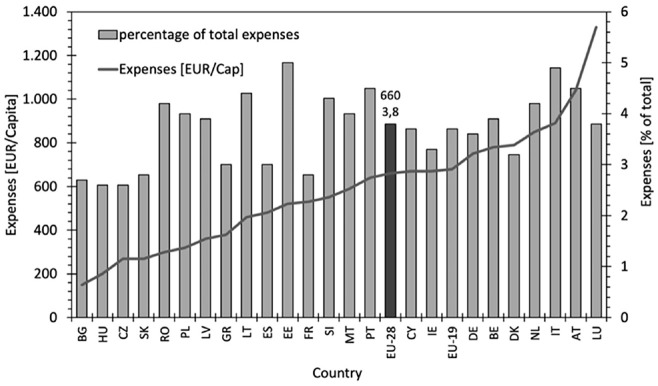
Final consumption expenditure of households for textiles in the EU (Eurostat, 2022).

The importance of the EU clothing and textile industry is reflected not least in the fact that 9% of its companies (i.e. 160,000 enterprises) and 5% of its employees (i.e. 1.5 million people) are located in this sector (EURATEX, 2020). In the EU, the household consumption of textiles amounts to 660 EUR per capita which corresponds to 3.8% of total expenses as shown in Figure 2. Even if the values within the EU vary significantly (between 2.6 and 5.0%, 150–1330 EUR per capita), the textile and apparel sector plays an important role in EU’s economy. In total, in 2020, EU’s textile and clothing industry generated a revenue of 162 billion € (EURATEX, 2020).

### The environment

Textiles and clothing manufacturing today plays a key role with regard to global climate change, water scarcity and environmental pollution in general. Over the last few decades, the production of textiles and clothing has increased tremendously and has been largely globalised (Boström and Micheletti, 2016). In 2019, the total production volume of textile fibres, the main constituents of textiles and clothing, already exceeded 100 million t (CIRFS, 2021) and has thus surpassed the production of the second-most important metal, Aluminium (65 million t (U.S. Geological Survey, 2022)). Between 1970 (22∙10^6^ t) and 2020 (101∙10^6^ t), fibre production has increased fivefold (CIRFS, 2021).

**Figure 3.**
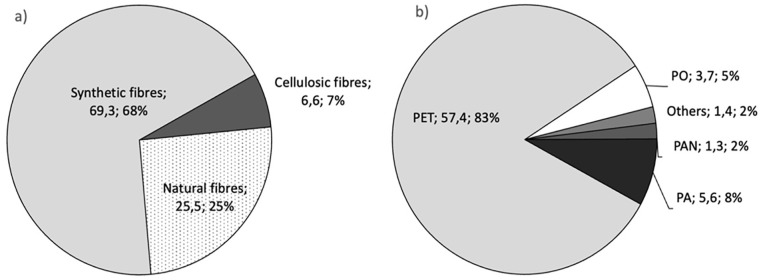
(a) Shares in total production volume of (man-made) synthetic fibres, (man-made) cellulosic fibres and natural fibres (mainly cotton 24.4∙10^6^ t and wool 1.1∙10^6^ t) and (b) shares of the synthetic fibre production in 10^6^ t, both in 2020 (CIRFS, 2021).

Basically, one can distinguish between natural and man-made fibres. The latter may consist of (fossil-based) synthetic polymers (of which Polyethylene terephthalate (PET) is of major importance) as well as (renewable) natural polymers (i.e. cellulose as the most important by amount). Figure 3 shows the production volumes of these fibre categories and their respective share in total fibre production.

**Figure 4.**
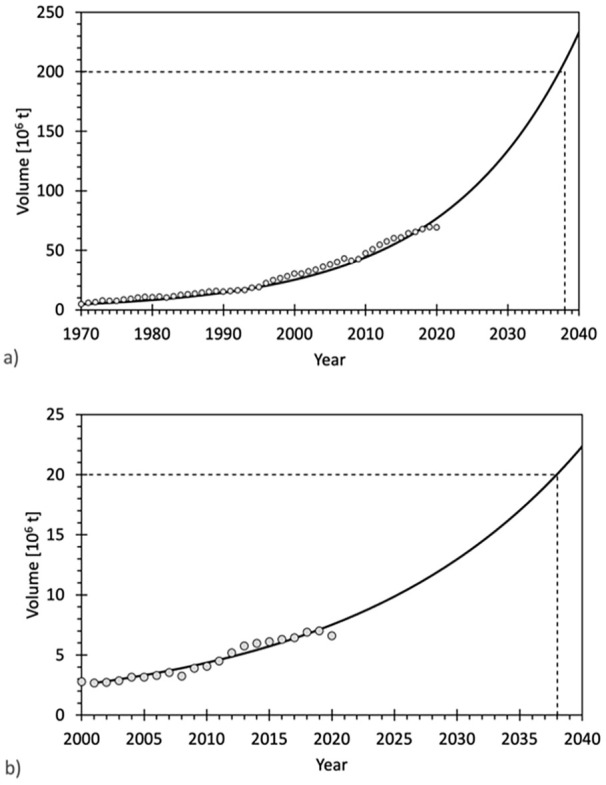
World production of (a) synthetic man-made fibres from 1970 to 2020 and (b) cellulosic man-made fibres from 2000 to 2020, (circles) and forecast (solid line) assuming the same growth as between 1970–2020 and 2000–2020, respectively (i.e. 5.6); (CIRFS, 2021).

The main growth driver are fibres from synthetic polymers, in particular PET, which increased from 4.8 (1970) to 69.2∙10^6^ t (2020), a fourteenfold increase (CIRFS, 2021). This corresponds to an annual growth rate of 5.6%. Assuming no changes in growth, the 200 million t mark would be reached in 2038 (Figure 4a). Cotton, as the second important fibre by produced amounts, has been stagnating over the last 15 years at an annual volume of around 25∙10^6^ t (CIRFS, 2021). The production of cellulose-based man-made fibres bottomed out in 2001 (2.7∙10^6^ t) and has grown by 5.5% annually since then reaching 7.0∙10^6^ t in 2019 (CIRFS, 2021). If this growth continues in the coming years, the 20 million t mark will be reached in 2038 (Figure 4b) and will then be close to today’s production volume of cotton. The production volume range of this fibre type, however, will remain in all likelihood at around 10% of the production volume of synthetic polymer fibres.

Table 1 shows the resource consumption and greenhouse gas (GHG) emissions to produce the main fibre types (PET, cotton and man-made cellulosic fibres). The production volume of these fibres was over 88 million t in 2020, which represented a share of 87% of global production. Based on these consumptions or emissions (related to 1 t of produced fibre material), the annual consumptions and emissions (for the year 2020) were determined. These consumptions and emissions to produce the respective fibre types are compared to the corresponding total global consumptions and emissions for fibre production in Table 2. It is found that fibre production accounts for about 1.4% of the world’s energy consumption, uses 7.5% of global water demand (this is mainly due to cotton cultivation and processing) and is responsible for 0.8% of the world’s GHG emissions.

**Table 1.** Resource consumption and GHG emissions for the PET, cotton and viscose fibres (Chapagain et al., 2005; Kalliala and Nousiainen, 1999; Shen and Patel, 2010; Shen et al., 2010). The numbers in parenthesis show the fluctuation range with different literature data.

Polymer	Production*	Energy		Water		GHG	
	10^6^ t	GJ/t	10^9^ GJ	m³/t	10^9^ m³	t CO_2_-eq/t	10^6^ t CO_2_-eq
PET	57.4	97 (95–98)	5.54 (5.45–5.62)	71 (17.2–125)	4.1 (1.0–7.2)	3.2 (2.3–4.1)	184 (132–235)
Cotton	24.4	58 (55–61)	1.42 (1.34–1.49)	12,100 (2000–22,000)	296 (49–542)	4.1 (3.7–4.5)	100 (90–110)
Viscose**	6.6	88 (70–106)	0.58 (0.46–0.70)	350 (300–400)	2.3 (2.0–2.6)	3.4 (1.5–5.3)***	22 (10–35)
Total	**88.4** ^#^		**7.5**		**302**		**306**

PET: Polyethylene terephthalate; GHG: greenhouse gas.

* By 2020 (CIRFS, 2021).

** Including modal and lyocell fibres.

*** Data do not include the deductions for biogenic carbon embedded in fibre (i.e. 1.4–1.7 t CO_2e_ per 1 t fibre).

# Corresponds to 87% of total fibre production.

**Table 2.** Resource consumption (energy and water) and GHG emissions in fibre production (considering 87% of global production, see Table 1) compared to the worldwide overall consumption and emissions.

	Energy [10^9^ GJ]	Water [10^9^ m³]	GHG [10^9^ t CO_2_-eq]
Global consumptions/emissions	556.6**	4009***	36.4****
Fibre-related consumptions/emissions*	7.53	302	0.306
Share of fibres	1.4%	7.5%	0.8%

PET: Polyethylene terephthalate; GHG: greenhouse gas.

* PET, cotton and cellulosic man-made fibres (88.4·10^6^ t), see Table 1.

** BP. (July 8, 2021). Primary energy consumption worldwide from 2000 to 2020 (in exajoules) [Graph]. In Statista. Retrieved February 14, 2022, from https://www.statista.com/statistics/265598/consumption-of-primary-energy-worldwide/.

*** FAO (Aquastat). (August 13, 2021). Total water withdrawals worldwide in 2020, by region* (in billion cubic meters per year) [Graph]. In Statista. Retrieved February 14, 2022, from https://www.statista.com/statistics/688173/global-water-withdrawals-by-region/.

**** Global Carbon Project. (November 4, 2021). Annual carbon dioxide (CO_2_) emissions worldwide from 1940 to 2020 (in billion metric tons) [Graph]. In Statista. Retrieved February 14, 2022, from https://www.statista.com/statistics/276629/global-co2-emissions/.

At first glance, the share of the fibre industry does not appear to be excessive; however, it must be considered that fibres are not the final product and a long and complex processing chain must be passed through to obtain the finished textile. Depending on the type of textile, especially the yarn count and grammage strongly influence the total energy demand, which can be even up to 960 GJ t^−1^, about ten times the value for fibre production alone (Ipsmiller and Bartl, 2022). Similarly, the GHG emissions for obtaining the textile can be significantly higher than those for producing the fibre and can be as high as 123 t CO_2_ t^−1^ (Nolimal, 2018). Based on these consumption and emission values, the importance of the circular economy in the textile sector becomes clear.

### The political framework

Until recently, textiles and fibres have not been in the focus of EU’s stringent waste legislation. In 2015, the European Commission (EC) announced the Circular Economy Package which did not mention textiles at all. However, in 2018, when the Directive (EU) 2018/851 (EC, 2018) amended the waste framework directive (WFD) (EC, 2008) textiles caught the attention of the European Commission. It is now clearly defined that textiles are explicitly part of municipal waste and that separate collection of end-of-life textiles must take place by 2025 at the latest (EC, 2015). Even if at present it is not yet determined which quotas will be applicable to collection, recycling or recycled content, it is foreseeable that the linear principle of the textile and clothing industry presenting a distinct source and sink will not be feasible to continue with. Reuse, life extension and recycling must become much more important in the field of textiles and clothing than they had been in the past.

In addition, the EC published the ‘Strategy for Sustainable and Circular Textiles’ on 30 March 2022 (EC, 2022). In the area of decreasing the environmental impact of textiles, it is no longer just about collection and recycling. Among other scopes, the EC announced the following measures, which can be considered very welcome as they lead in the right direction:

- Introducing mandatory ecodesign requirements for newly developed textiles
- Stopping the destruction of unsold or returned textiles
- Tackling microplastics pollution to the environment
- Introducing information requirements and a digital product passport for better transparency and traceability
- Green claims for truly sustainable and eco-friendly textiles
- Introduction of extended producer responsibility (EPR) and boosting reuse and recycling of textile waste

It is clear that while the intended targets could significantly reduce the environmental impact of the textile industry, the document lacks approaches, as to how and by which exact means these targets can be achieved. For example, it does not outline how EPR for textiles will be implemented. It is merely announced that the waste framework directive will be amended again in 2023 and it can be expected that only then the EPR system for textiles will be more clearly defined. It can be concluded that EU legislation in the field of textiles is in a state of flux. The EC wants to significantly reduce the negative environmental impact of this industry but has not agreed yet on how this should be done.

### The technology

At first glance, one might assume that textile recycling was quite easy. PET represents the most widely produced fibre material for textiles (57.4∙10^6^ t, i.e. 57% market share (CIRFS, 2021)) and is – in principle – the same polymer as used for beverage bottles. It is reported that in Germany the recycling rate of PET bottles in 2019 was as high as 92% (bvse, 2020). On the other hand, 31 million t of textile fibres (31% market share) are composed of cellulose (cotton 24.4∙10^6^ t and cellulosic man-made fibres 6.6∙10^6^ t) which is the basic material of paper and cardboard. In comparison, the recycling rate of paper amounts to 85% in Austria (ARA, 2019).

In contrast to PET bottles and paper, about two-thirds of textiles end up in residual waste while a ‘high-quality fibre-to-fibre recycling is virtually non-existent’ (EEA, 2021). One reason for this is the fact that textiles frequently consist of two or more fibre types within one item. Even though PET and cellulose are recyclable as pure materials, they are incompatible for state-of-the-art recycling processes when mixed. In addition, additives such as dyes, surface finishes and the like make recycling processes even more difficult. Adding to that the mentioned long textile processing chain must be considered which is the reason for different recycling processes for application at different stages are being studied or have been used to some extent, as explained in more detail in Table 3.

**Table 3.** Principal categorisation of textile recycling processes.

Type of recycling	Principle	Comment
Recycling on fibre level	Mechanical disintegration of fabric into individual fibres	Dependent on input material a blend of different fibre types and colours is obtained resulting in low quality frequently called mechanical recycling
Recycling on polymer level	Re-spinning of fibres by melt extrusion (e.g. PET) or solvent spinning (e.g. viscose)	Only viable for pure input materials frequently called chemical recycling
Recycling on monomer level	Breaking down the polymer into monomer	Savings potential relatively low as the complete textile processing chain has to be redone (not considering certain benefits by side-product refining when such streams occur during other types of recycling processes)

A mechanical separation of the different fibre types is in most cases not possible. In woven fabrics, warp and weft can be made of different yarn materials. Hence, application of such processes will lead to the recovery of fibre blends, which are undesired in most cases – exceptions being the interest of current research. Things can get even more complicated when fibres made from different polymers are blended one level below within the yarn itself, which will clearly render a material separation impossible (Redmore, 2020). Moreover, and equally an issue with single-material textiles, there is the problem of the generally dense structures of textile fabrics that will lead to high mechanical force at tearing, which is conflicting the recovery of fibres with the desired quality characteristics. Recycling of single material textiles, however, such as pure PET or pure cotton, is already achievable using different technologies. For instance, cotton cuttings from apparel production can be used to manufacture lyocell fibres (Kaserer, 2020). This procedure is already viable on an industrial scale (https://www.tencel.com/de/refibra). For textiles made of two polymers, one promising route is a (bio)chemical removal of one of the two components, making the remaining, now pure, component recyclable. For example, enzymatic hydrolysis can be used to break down cotton fibres into glucose in an aqueous solution, whereas the remaining PET can then be used as a raw material for a fibre spinning process. The recycled fibres can be further processed into textiles (Piribauer et al., 2021). However, the process is not yet realised on an industrial scale and a future industrial realisation also needs to consider the removal of (functional or process) additives and contaminants.

This paper presents an extensive and comprehensive overview of textile recycling and the related challenges based on the latest published scopes and legislative measures by the EU in 2022. The focus on textile recycling induced by recent and expected legislature, establishes a strong mandate to increase textile recycling. Likewise, the awareness within industry and expressed in consumer behaviour has been low or repressed until now and is therefore due for change. A consultation to players in the European textile industry highlights the importance of a more circular fashion system not only in the EU, but also on a global level.

## Methods

Fibre-to-fibre recycling is an upcoming strategy in the apparel sector and currently little information is available regarding capability, prerequisites and necessity. Also, the expression is sometimes ambiguous. Therefore, in this work, the market situation and the economic boundaries for fibre-to-fibre recycling are evaluated. An assessment of the apparel fibre market has been conducted in cooperation with stakeholders from the European clothing industry. A survey invitation has been accepted by four European fashion companies and one European textile recycling organisation; the survey can be found in the Annex. The fashion brands are sellers of sportswear, jeans and casual clothing. After finalisation of the survey, the outcome has been intensively discussed in a follow-up discussion round for detailed gathering of the stakeholder’s expertise. The focus of the discussion round was the sourcing of fibres, the current market situation of textile production and the economic boundary conditions as well as the effect on the upcoming fibre-to-fibre recycling methods.

## Results and discussions

### Primary fibre sourcing

A common way of sourcing fibre materials for production input is from new resources or so-called primary resources. Globally, the most produced fibre by far is PET ((Figure 3) with a volume of 57.4∙10^6^ t in 2020 (CIRFS, 2021). The share of recycled PET in fibre production is around 14%, whereat recycled PET (rPET) is virtually exclusively derived from collected PET plastic bottles (Textile Exchange, 2021). Stakeholders import PET fibres as virgin material or recycled form from Asian countries like China, India, Vietnam and Taiwan, whereat Spain was the only European country mentioned. These statements seem reasonable, especially since the European production of PET fibres in 2020 was only 1.17∙10^6^ t (i.e. 2.0% share; CIRFS, 2021).

**Figure 5.**
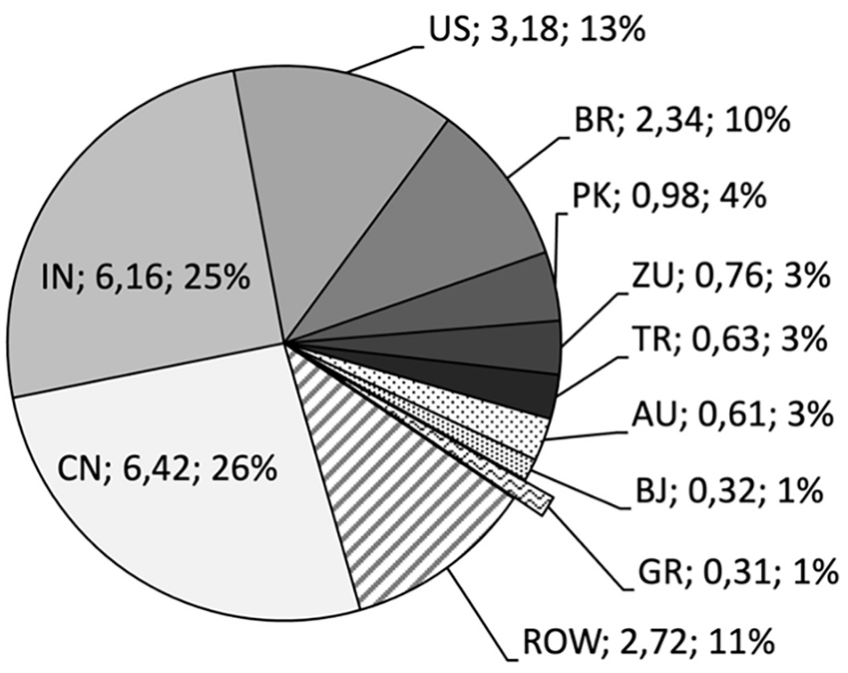
Leading cotton producing countries worldwide in 2020/2021; Country abbreviations according to ISO 3166-1; ROW: rest of the world; production volumes in 10^6^ t and respective share in % (U.S. Department of Agriculture, 2022).

Cotton marks the second-most important material in global fibre production, with a production volume of 24.4∙10^6^ t in 2020 (CIRFS, 2021), with a share of 0.26∙10^6^ t (i.e. 1.06% only) of recycled cotton (Textile Exchange, 2021). Most stakeholders source this fibre type from Asian countries and mentioned China and India. This is comprehensible, since China and India alone hold a share of 51% of the global fibre production (see Figure 5) whereas the share of Greece, as the most important EU cotton producer, is only 1.25% (i.e. 310∙10^3^ t).

### Secondary fibre sourcing via fibre-to-fibre recycling

**Figure 6.**
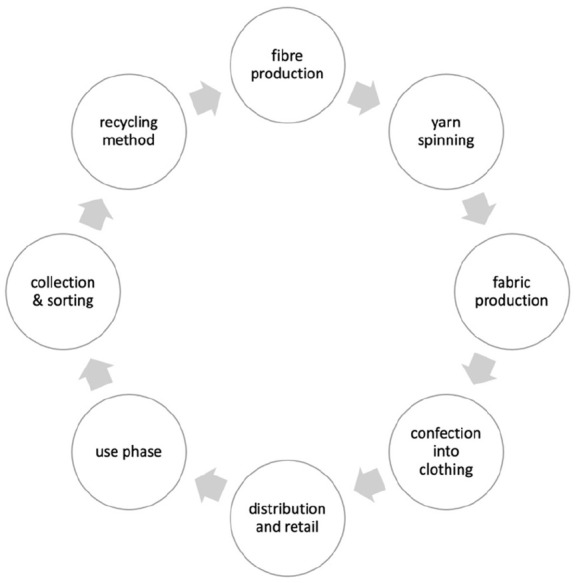
Scheme of fibre-to-fibre recycling.

As already mentioned above, separate collection of textile waste will be compulsory in the EU by 2025 (EC, 2018). This upcoming legislation will certainly show a huge impact on quality and quantity of collected textiles. On the one hand, more textiles will be collected and, on the other hand, the share of non-wearable (i.e. to be recycled) goods will increase. The increased volume of textiles that need to be recycled poses major challenges for the recycling industry, but also brings opportunities to increase sustainability in the sector and provide secondary raw materials. It is already common practice to recycle textile fibres for different applications such as filler material for insulations or mattresses (Girn et al., 2019; Rotimi et al., 2021). However, this type of recycling currently results in rather low-quality products and can be considered a cascade utilisation. Nevertheless, recycling at low quality levels mostly has advantages over thermal recovery. Incineration is a common practice in some European countries, especially when landfilling is already prohibited (e.g. in Germany and Austria) and is in any case preferable to landfilling (Manshoven et al., 2019; Ellen MacArthur Foundation, 2017). An upcoming approach is the reprocessing of fibres within textiles into new fibres via re-spinning (i.e. fibre-to-fibre recycling) which tackles the problem of huge amounts of discarded textiles (Girn et al., 2019). In Figure 6, a rough scheme of typical fibre-to-fibre recycling is presented.

In this context, it is important to mention that compared to other products, such as PET bottles or paper, the manufacturing process for textiles requires a multi-stage process chain. There are therefore several possibilities for feeding materials back into the manufacturing process, namely as fibres, as polymers or as monomers. As explained in detail elsewhere (Bartl, 2019; Bartl, 2020; Ipsmiller and Bartl, 2022), the less far back in the production chain it is necessary to go, the greater the potential for resource savings. Nevertheless, there is no such thing as the optimal recycling process, but it will be necessary to apply a set of different processes depending on the type and condition of the end-of-life textiles, in order to achieve the best possible environmental protection, benefit and the highest economic efficiency. Regardless of the technology, this paper seeks to explore the causes of the currently low recycling rates.

**Figure 7.**
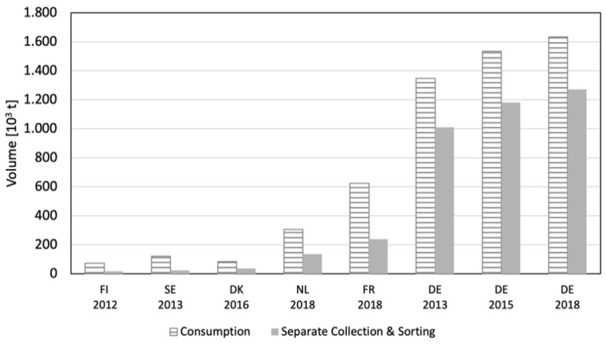
Textile Consumption and Separate Collection and Sorting in EU member states in kt; Figures adapted from (Watson et al., 2020) and (bvse, 2020).

An indispensable step and prerequisite for a feasible fibre-to-fibre recycling is the separate collection and sorting of textile waste. Even though separate collection of end-of-life textiles is of eminent importance, European waste legislation has not yet attributed much importance to this waste stream. Up to now, collection schemes for textile waste in the EU are based on voluntary initiatives. Charity organisations as well as municipalities and commercial enterprises are active in this field (Manshoven et al., 2019). Collection and sorting are funded by the sales of second-hand clothing. As a matter of fact, collectors demand reusable items only and are not motivated to gather goods that are only suitable for recycling. The only exception is France where a scheme of EPR has already been introduced in 2009 (Re_fashion, 2020). Figure 7 shows selected EU member states, in which collected and sorted textile waste volumes have been monitored. Compared to the consumption of clothing, the collection rates are still low. Referring to figures for Germany, which is far ahead of the other countries, a further rise in apparel collection and sorting could be seen over the last few years.

However, based on new EU legislation (Directive (EU) 2018/851), a separate collection of textile waste will be mandatory latest by 2025. Even if until today no targets or quotas have been announced by the EC, a massive change in the handling of end-of-life textiles can be expected.

### Challenges in fibre-to-fibre recycling

Commercially and fully developed technologies for economic and ecological fibre-to-fibre recycling are not yet available. Different recycling technologies are still in development and further validation on laboratory and pilot scale is being conducted (Leal Filho et al., 2019; Duhoux et al., 2021).

#### Lack of collection and sorting systems

An indispensable prerequisite for fibre-to-fibre recycling is a sufficient amount of processable discarded textiles. Today, not enough textile collection and sorting systems are operating to fulfil the needs of ‘emerging textile recycling organisations as discussed before’. A significant portion of the amounts collected is deemed non-usable, because discarded textiles are often stained, soiled or torn apart (Girn et al., 2019; Watson et al., 2018). The revised EU Waste Framework Directive ensures that EU member states will need to introduce an obligatory collection of discarded textiles (EC, 2018), so it can be assumed that the amount of collected discarded textiles will increase in the future.

#### Necessary research in collection and sorting technologies

Textile waste collection and sorting systems principally operate in two stages. Stage one is the distinction into reusable, recyclable and unusable fractions. After that, the second stage aims at separating the different materials present in recyclable fractions depending upon composition. Today, this is done via manual sorting or technology-enabled manual sorting with a differing degree of automation. Stakeholders as well as Köhler et al. (2021) mention that manual sorting is accurate but very expensive due to labour costs. Sorting staff need good identification skills regarding the composition of the discarded textiles. A great reduction in sorting expenditures by including automated sorting systems is necessary to feed upcoming fibre-to-fibre recycling methods (Girn et al., 2019).

One promising method for fully automated sorting and appreciated by stakeholders is an optical technology for on-line capture of the textile composition. Several organisations and publications are focusing on the promising near-infrared technology (Girn et al., 2019; Duhoux et al., 2021; Re_fashion, 2020; Roithner et al., 2021). Although automated processes are preferred over manual sorting, they are still on pilot scale or in development. In the near future, with the aforementioned rising textile waste volumes that can be expected, an industrial scale must be achieved.

#### Composition and condition of collected textiles

Textiles have to fulfil requirements regarding functionality, durability and wearing comfort which frequently makes it necessary that they consist of two or more different fibre materials (Harmsen et al., 2021). As already mentioned in Section ‘The technology’, this complex composition of materials within textiles makes them difficult for recycling. The stakeholders highlight that wearing comfort of apparel is the most important driver for multi-material textiles.

**Figure 8.**
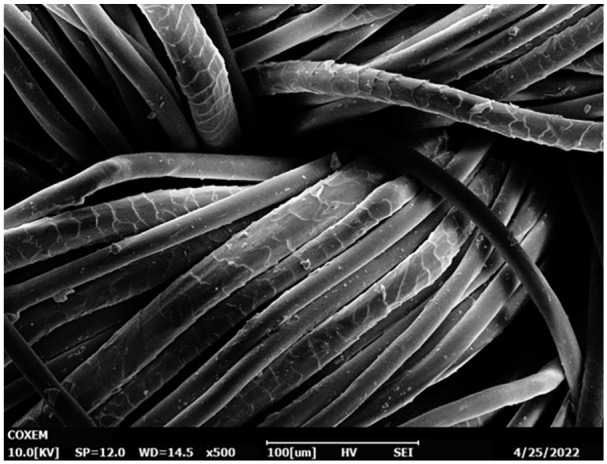
SEM image of a wool/PET/elastane trousers.

Figure 8 presents an example of a typical mix of fibres in apparel. The image gathered via scanning electron microscopy (COXEM EM-30 plus, COXEM, South Korea) shows a morphological detail of a ladies’ pants. These trousers are labelled with following contents: 53% PET, 44% wool and 3% elastane. The wool fibre with its typical roof-tile-shaped surface can easily be recognised. The PET fibre can be identified with its smooth surface and transparent appearance, but it is hardly distinguishable from an elastane fibre which has the same appearance within the used imaging technique. The diameter of the fibres ranges between 15 and 20 µm, which ranges within the typical dimensions. Both fibre types are intensely commingled and it is obvious that a mechanical separation is not a proper method for an accurate separation.

However, textile products moreover consist of trims, zips and badges, which are mainly made from different polymers or metals (Leal Filho et al., 2019). Additive chemicals are also used with apparel for giving the apparel a different appearance or property (Chen et al., 2021; Duhoux et al., 2021). Examples for this are adding colour by pigments or using organic compounds such as flame retardants that are spun into the fibre (Vecchiato et al., 2018).

The range of the discarded textiles’ condition tends from clean, rarely used products to stained, oil-soaked and torn fabrics that cannot be reprocessed. One stakeholder makes use of a sustainable circular material procurement by collaborating with a local social company and explained this circumstance. They collect and manually sort cotton textile waste to reprocess it in apparel production. Only cotton textile waste in good quality condition, that is, without any soiling or grave physical alteration, can be used as production input. Cotton textile waste in excellent condition is separated and provided for a local thrift shop and non-reusable fractions are shredded into smaller pieces and function as filling material for car seats or building materials.

Elastane in textiles affects the recycling process (Jönsson et al., 2021). As a result of the chemical structure, textiles containing elastane in larger amounts are not yet suitable for state-of-the-art recycling methods, where a limit may already be reached at a sub-1%-level. The rubbery material cannot be easily decomposed thermally, and it tends to clog filters of recycling machines, resulting in a process downtime because of the rising pressure and the necessary cleaning associated with it, let alone possible permanent damage. When textiles are cut into small pieces and are fed into tearing lines, an elastane content of more than 3% is a problem and creates white nodules in the fabrics after remanufacturing. Up to now, visible light (approximately 380–750 nm) and infrared light (approximately 0.75–1000 µm) detectors have reached their limits when it comes to identifying and quantifying elastane content in textiles, as the individual fibre types might cover each other (see Figure 8) or sheath-core yarns are used. With the low penetration depth of the used radiation, covered regions will not be detected. One promising method is the previously developed elastane quantification tool (Boschmeier et al., 2023). An accurate and fast quantification of elastane shares is possible, albeit not contactless, which facilitates textiles recycling containing elastane.

#### Location of textile waste collection

Stakeholders agree that the collection and sorting stage for material recycling is very important and introduced a significant criterion: the location of textile waste collection. They point out that the location of collection has a huge impact on the quality and quantity of the discarded textiles. Stakeholders mentioned four locations with their advantages and drawbacks:

(1) At a retailer’s store: some big fashion companies already offer in specific stores the acceptance of discarded textile waste. Stakeholders highlight that these discarded textiles are in excellent condition for the most part. Usually, consumers bring their clothes that do not fit or match anymore or they are just old-fashioned in their opinion.
(2) In/next to citizen’s homes: alternatively, instead of disposal in the usual trash bins, citizens could discard of their textile waste by taking it to an acceptance place such as, for example, a recycling centre. Stakeholders point out that the quantities are very low today, but the quality is indeed very high.
(3) In urban kerbside collection: charities and non-profit organisations use this collection form across EU cities, but stakeholders have no expertise in the textile quality.
(4) Central collection places in the form of big containers: stakeholders agreed that this location is a preferred one, since the industrial handling is easy, and they assume that implementation is not difficult. However, stakeholders explained that practical experience showed that the quantity of this collection form was very high, but that quality was rather low. Dirty, stained, and oil-soaked discarded textiles would find their way into these big containers and might pollute textiles in good condition within the collection vessel.

### Economic boundary conditions

During the discussion round, stakeholders aimed at pointing out legislative requirements for the European textile industry to achieve the sustainability goals and keep up the competitiveness in the market.

### Certification

Stakeholders are aiming at more intense use of certificates serving as sustainability standards. Certificates themselves aim at increasing recycled content in products and reducing the environmental impact caused by production processes. Fashion brands seek for a certain amount of trustworthy and independent certificates. Certificates used in textile industry today and appreciated by stakeholders are Global Recycling Standard (Textile Exchange, 2020), Global Organic Textile Standard (Global Standard GmbH, 2020) and OEKO-TEX (OEKO-TEX Association, 2021). Certificates ensure that the textile value chain operates under controlled conditions. Stakeholders also approve that the number of certificates ought to be limited, for too big an offer of certificates would create an information overflow to the consumer, counteracting the purpose.

A legislative framework is needed for a mandatory use of certifications in fashion products, as there is currently no regulation requiring fashion brands to use certifications for their products. The sourcing of environmentally sustainable raw materials without making use of child labour is among the stakeholders’ most mentioned issues. Eventually, all brands agree that they need generally mandatory tools at hand that would provide guarantee for certain standards.

### Fast fashion

Fast fashion stands for a business model based on high production volumes, presenting low-quality products with short market launch cycles that are frequently also sold for cheap prices. As such, the dwell time on the market for fast fashion products is fairly low (Yalcin-Enis et al., 2019). The idea of this business model is to keep up with the latest design trends by bringing new collections of apparel on the market in short periods. In this way of thinking, the consumer is able to always buy the latest trend at low prices, resulting in a mass consumption of low-quality products (Manshoven et al., 2019). Big Fast Fashion brands are bringing 16 or 24 collections on the market within 1 year (bvse, 2020). Stakeholders explain the heavy impact of fast fashion on the apparel market and less resulting room for circular and sustainable fashion that is currently more expensive. Companies that focus on sustainable products have to deal with the handicap of not being competitive on the market right now. Or, to quote one stakeholder:

It can’t be the case that consumers and brands that make the right choices, are financially punished for this.

### Landfill and export of textile waste

Nowadays it is common practice that collected and sorted textile waste is landfilled or incinerated, as there are too few other areas of application (Rotimi et al., 2021). Not enough well-developed circular solutions for further processing are in existence and already existing ones are challenging (Köhler et al., 2021). Besides the lack of suitable recycling technologies and alternative post-use treatment routes for textile waste other than landfilling, the issue is aggravated by another fact: the export to other countries (Manshoven et al., 2019). By following their business models, brands as well as countries have different reasons to exporting textile waste, like (Bartl, 2020; Ellen MacArthur Foundation, 2017; Manshoven et al., 2019; Repp et al., 2021; Watson et al., 2020):

- High labour costs in well-developed countries. By exporting textiles to low-income countries such as in Africa or Asia, the costs are lowered
- Generating revenue: selling collected and sorted clothes is a business
- Ban on landfilling of textile waste: some EU countries have a ban on landfilling and others do not. The ones with a landfilling ban in place are sending large amounts of textiles to other countries, even within the EU. Concerning European countries’ waste, exported textiles tend to be landfilled abroad (frequently ending up in non-sanitary landfills or open dumps), which means the problem is not solved but made into a global problem

Stakeholders also provided information that currently, no strict regulations are applied on which textiles are exported and on where they are exported to. Exporting textiles has been a growing market activity across Europe. In 2019, the export rate of textile waste from the EU-27 countries was about 1.3 10^6^ t, whereas in 2003 it was 400,000 t (Köhler et al., 2021).

### Legislative instruments

If it is not possible to produce sustainable textile material at the same costs as in the ‘normal way’ using virgin materials, instruments ought to be created that facilitate the market uptake of recycled textile products. Stakeholders agree that using the European Green Deal idea to a modern, resource-efficient and competitive economy encourages textile recycling. Financial, legislative and social instruments need to be put in place to create the most important thing: market pull for recycled fibres. Appropriate actions will result in higher request for recycled fibres, further developments in recycling technologies and pressure on fashion brands to develop apparel containing recycled material. As a result, the snowball effect will also push the various fibre-to-fibre recycling approaches. All stakeholders agree that in the world today there is a need for a system where sustainability is pushed forward and supported. Legislative instruments that support sustainability and a better future within the textile industry need to be put in place. Within the discussion round, stakeholders agreed on the following key policies:

#### Mandatory certification for textiles

One starting point for ensuring a sustainable future and driving the textile sector in the EU to a genuine circular economy, is a mandatory certification for produced or imported textiles. Textiles have to be labelled with certificates, so that the consumer is aware of buying sustainable clothing. In order for that to be reliable, supervisory authorities should be monitoring the certification system.

#### Import ban on low-quality apparel

Among the stakeholders, the conclusion was drawn that an import ban on low-quality apparel should be put in place. Setting minimum standards for textiles for certain technical criteria such as number of minimum washing cycles a piece needs to endure and stay in shape is required. A directive is needed to deaccelerate fast-fashion trends.

#### Mandatory education on sustainable lifestyle

Stakeholders explain how young people in the age between approximately 10–20 years try to ‘find themselves’ and experiment with different clothing styles. At this point, influence can be generated in a positive way. Today’s young generation is not aware of the impact they have when buying cheap goods such as frequently fast-fashion clothing items. On the other side, consumers do care about the environment, climate change and the planet’s future (Rotimi et al., 2021). The next generation need to be made aware on how they can change the world by encouraging them to act differently in front of the grown-ups.

However, education has its limits: people intend to buy clothes as a reward, to fit into a social group or to stand out within their friends, as stated by a stakeholder. Education in schools and with big players in the fashion industry needs to start challenging the younger generation soon. Education institutions shall be addressed to place a sustainable lifestyle as one of their core-topics and fashion brands should enhance this mind-set. Within this new education form, parents shall also be considered. A conscious consumption must be achieved in the near future.

#### Textile waste export regulations

Discarded textiles contain large amounts of raw materials that could be recycled. High percentages of discarded textiles are exported to other countries. Regulations could keep the material inside the EU and push the industry to make use of it.

## Conclusion

In order to run along with the European Green Deal to a competitive and circular economy, the reprocessing of textile waste is indispensable. Today, large amounts of discarded textiles are either landfilled, incinerated or exported to countries abroad. These common uses are a threat to a sustainable, modern textile sector. The revised EU waste framework directive (Directive EU 2018/851) stipulates a mandatory separate collection of textile waste for EU member states by January 2025. Although the collection and sorting process for discarded textiles is not easy, more locations for efficient textile collection need to be created by EU member states. It is important to consider different aspects of the collection places, because experience shows that the quality of discarded textiles differs between central places, kerbside, household and retailer collection. Apparel consists of different woven or knitted and/or otherwise jointed materials that are hard to separate. Further research and development in sorting technologies is an absolute must for an accurate separation of the different materials used in apparel and subsequently, increasing technical accessibility of recycling processes for those materials.

The idea of fibre-to-fibre recycling seems a promising set of technologies for the future. Yet, the easy principle of using discarded textiles as production input for new textiles comes with some quite not so easy challenges. As soon as the background of an essential single variety textile material supply without soiling is established, a more suitable input for fibre-to-fibre recycling is available, resulting in more focus on developing this recycling technology further. A facilitation of the market uptake of recycled fibres and its economic boundary conditions were discussed intensively. Some companies aim at using more recycled materials to get in line with their sustainability goals, so a slight market pull can be assumed, yet market competition is hampering the adoption to a greater extent. Strict key polices by EU legislature such as import bans on low quality apparel and export regulations on textile waste can best guarantee that feasible material for certain recycling technologies stays in the European Union. Mandatory certification on new textile products will ensure more sustainable products and lead to pressure on the fast-fashion business model that stands for low quality products, cheap prices and high production volumes that largely counteract efforts by forerunners for a more sustainable textile sector. The fast-fashion business model is contrary to the EU Green Deal as well, yet actually contemporary to a certain degree among young people’s mind-sets. Education on sustainable lifestyle needs to be taught more intensely in schools to encourage the young to better resource usage and shopping behaviour, because they make up the largest target group for fast fashion and may thus play a key role in a future sustainable textile industry.

## Annex

### Survey 27th August 2021

#### Area 1: Recycled fibres

1. To which point are you involved in the value chain of recycled fibres?
2. Which products exist in your market segment, that contain a certain percentage of recycled fibres?
3. What forms of recycling of cotton, PET, PA, EL and viscose (cellulose regenerate) fibres are used in your region/business/products?
4. What are common problems of recycling the five above mentioned materials (including blends) in your region/business/products?

#### Area 2: Technical requirements (mechanical, optical, haptic)

1. Which are the technical criteria (parameters) of used fibres that are important for your products or your field of research?
2. Could you specify the latitude or range those parameters need to attain to be used in the production process?
3. Which experiences do you have in terms of purchasing recycled fibres (domestic, abroad)?

#### Area 3: Facilitate market uptake

1. What’s the cost gap between recycled and primary fibre of the five mentioned materials in your region/business/product?
2. If you already use recycled fibres: what is the cost gap of recycled fibres from the sources you know (e.g. domestic, import from low wage countries)?
3. What are the economic boundaries, that hinder the market uptake of recycled fibres?
4. What are consumers ready to pay and at which level is the loss of margin, or which loss would be acceptable in your company’s/brand’s opinion, respectively?
